# Injection-induced fault slip assessment in Montney Formation in Western Canada

**DOI:** 10.1038/s41598-022-15363-8

**Published:** 2022-07-07

**Authors:** A. Yaghoubi, M. B. Dusseault, Y. Leonenko

**Affiliations:** 1 Department of Earth and Environmental Sciences, University of Waterloo, Waterloo, ON Canada; 2 Waterloo Institute for Sustainable Energy (WISE), University of Waterloo, Waterloo, ON Canada; 3 Department of Geography and Environmental Management, University of Waterloo, Waterloo, ON Canada

**Keywords:** Structural geology, Geophysics

## Abstract

Hydraulic stimulation to enhance energy extraction from geothermal and unconventional resources is typically accompanied by seismicity because injection changes pore pressures and temperatures, facilitating slippage of fractures and faults. Induced seismicity carries potential risk if events are large enough to damage infrastructure. The uncertainty invariably associated with the state of stress measurements and subsurface geomechanics parameters affects the analysis of fault slip and seismicity induced resulting from hydraulic fracturing. In this study, a probabilistic approach is used to assess the slip tendency of known faults crossing the compartmentalized Montney Formation of western Alberta and northeastern British Columbia. We first divide the formation into four different stress areas based on pore pressure deviations from hydrostatic. In each stress area, geomechanics parameters are expressed as probability distributions using multivariable datasets from borehole petrophysical data to injection-induced focal mechanisms. Monte Carlo simulations are applied to assess the potential slip tendency of local faults. We display the cumulative distribution function of critical pore pressure to cause slip on each fault by using analyses of the parameters of the Mohr–Coulomb shear failure criterion and local tectonic stress state. The results provide useful input for seismic hazard assessment and risk mitigation for local faults affected by high-rate fluid injection.

## Introduction

The Montney Formation, a prominent unconventional shale gas and liquids resource, covers approximately 130,000 km^2^ in northwest Alberta and northeast British Columbia. The area is one of the most productive unconventional hydrocarbon resources in the Western Canada Sedimentary Basin (WCSB). According to a 2013 study by the British Columbia Oil and Gas Commission and the Alberta Energy Regulator, the Montney Formation can produce 12,719 billion m^3^ of marketable natural gas, 2,308 million m^3^ of marketable natural gas liquids (NGL), and 179 million m^3^ of marketable oil^1^. By the end of 2019, more than 3600 wells had been completed in the Montney Formation just in British Columbia^2^.

**Figure 1:**
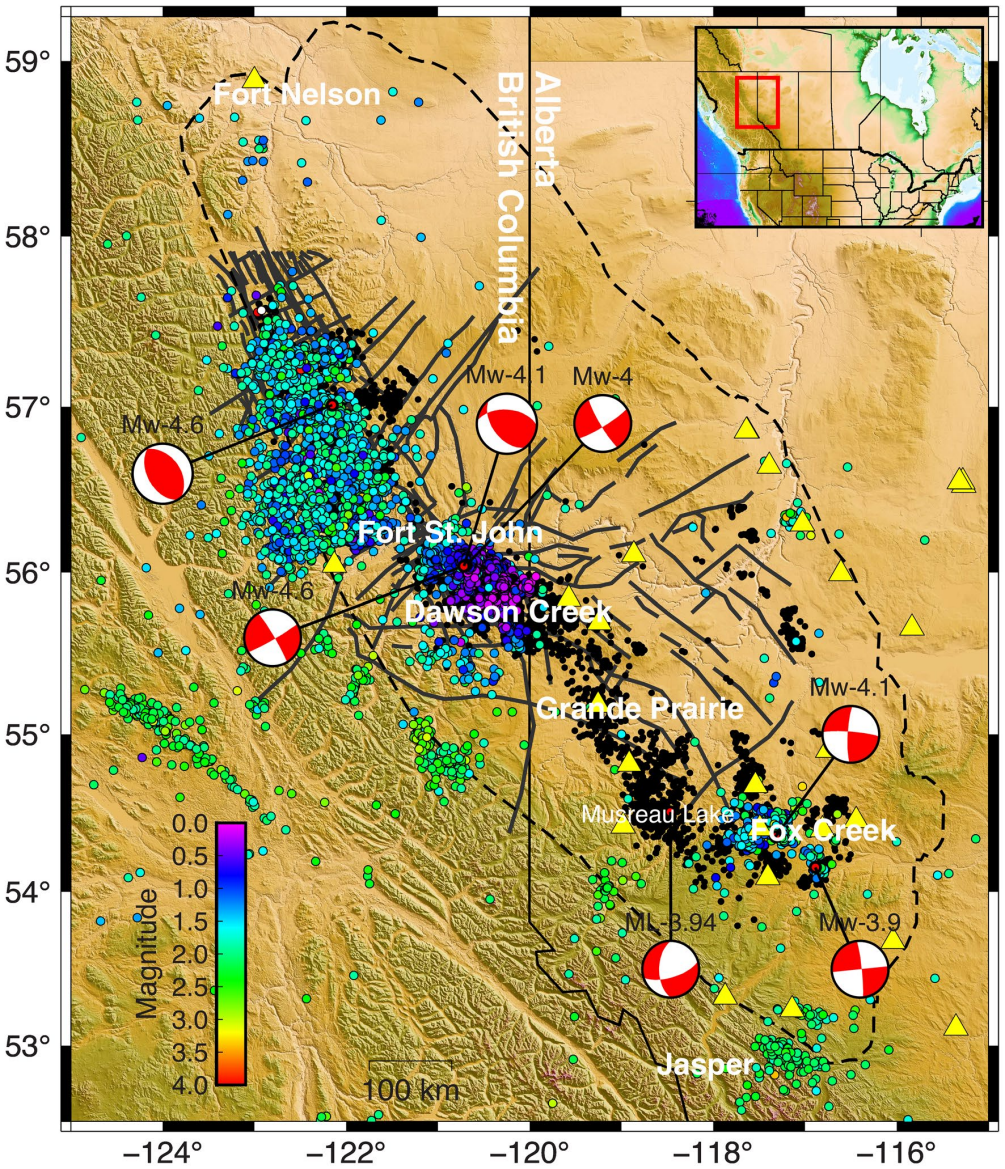
Seismicity, fault traces, HF wells in the Montney Formation. The dashed line defines the Montney Formation area. The colored circles are seismicity reported by Visser et al.^34^ and Visser et al.^8^. Not all these earthquakes are within the Montney Formation; the colored circles outside of the area of the Montney Formation are natural tectonic earthquakes. The earthquakes around Fox Creek have resulted from HF in the Duvernay Formation and wastewater disposal near Musreau Lake (event ML 3.94)^35^. Grey thick lines are the main faults in the studied area. Black dots show wells drilled into the Montney Formation. Geographical locations of seismic stations are indicated by yellow triangles^36^. The mechanism events represent some major earthquakes recorded in the area. The figure was generated using the Generic Mapping Tools (GMT) V.6^37^.

Shale gas and shale oil production from the Montney play has grown with the use of multi-stage HF (Hydraulic Fracture stimulation) technology. Supported by high oil prices and new HF technology availability, development started in 2005 and accelerated significantly in 2011; since then, the seismicity rate has increased^3–6^. More than 200 M_W_ > 3 earthquakes within the area 52° N–60° N and 114° W–126° W are spatiotemporally associated with HF operations (see Figure S1 in Supplementary Information). In the last decade, noticeably higher seismicity rates have been observed in previously quiescent areas of British Columbia and western Alberta (Fig. 1)^3–6^. The anthropogenic seismicity for this area includes some of the largest M_W_ values reported globally, including events near Fort St. John of M_W_ 4.6 on August 17, 2015^5^, and M_W_ 4.2 on November 30, 2018^7^. Most of these occur during HF treatments and are spatially and temporally restricted to the region around the wells^4^. M_W_ 4.6 on August 17, 2015, for example, occurred after five days of fluid injection of 65,000 m^3^ in the Lower Montney Formation (depth of 1.9 km)^5^ (see Figure S2 in Supplementary Information). The area between Fort St. John and Dawson Creek, BC (the Kiskatinaw area) and the northern Montney trend are the seismogenic regions within the Montney depositional area^8^ (Fig. 1).

HF operations have not always resulted in injection-induced seismicity in the study area. Induced seismicity clouds show a high likelihood of slip due to HF in the area around the Kiskatinaw area as well as in the northwestern Montney area. Even in these areas, not all HF stimulation activities were associated with induced seismicity^4,6^. Atkinson et al.^4^ highlight that of 12,289 wells drilled in the WSCB between 1985 and 2015 and hydraulically stimulated, only 0.3% were associated with injected-induced earthquakes of M ≥ 3. A subsequent study by Ghofrani and Atkinson^6^ determined that 0.5 to 1% of HF wells drilled in WCSB between 2009 and 2019 were associated with induced seismicity M ≥ 3, which indicates that the rate has steadily increased over time. They have observed that the associated rate of M ≥ 3 earthquakes appears to be formation related; the Montney Formation has an associated rate of %2 whereas that rate for the Douverny Formation is 6% and that of others is much lower. Questions arise as to which mechanisms are responsible and what parameters control regional injection-induced seismicity in the study area.

Three mechanisms can be considered for injection-induced seismicity. First, increasing pore pressure during HF decreases the normal effective stresses acting on fault/fracture surfaces, inducing shear slip, and causing earthquakes^9–12^. Second, coupled matrix poroelastic effects during HF in a fractured rock cause stress changes. Therefore, slippage/earthquakes may occur, perhaps not directly related to an increase in local pore pressure, but sufficient to trigger slip along a critically stressed discontinuity^13–16^. Deng et al.^13^ performed a fully coupled poroelastic simulation to evaluate the spatiotemporal changes of solid matrix stresses and their relation to the 2013 Crooked Lake seismicity sequence in central Alberta. Their results showed that the poroelastic mechanism is responsible for both delayed and immediate injection-induced seismicity^13^. The same causative mechanism has been proposed by Wang et al.^14^ and Konstantinovskaya et al.^17^ to account for injection-induced seismicity in WCSB. Third, cases may arise in which faults can very slowly transform into a slippage state during HF, and fluid injection triggers aseismic (stable) slip^18–20^ sufficiently far from the reservoir depth. Studies by Eyre et al.^19^, Yu et al.^18^, and Eyre et al.^20^ suggest that induced earthquakes in WCSB may be attributed to aseismic slip loading. Another study^16^ suggests a combination of direct pore pressure diffusion and poroelastic stress changes as the possible mechanism behind injection-induced seismicity in the Montney Formation. For this current study, the assessment of injection fault slips is based on the first mechanism: fluid injection causing normal stresses to decrease within the fault plane, which in turn, destabilizes the fault.

The magnitude and rate of anthropogenic earthquakes are influenced by two sets of field parameters directly: the controllable operational parameters, including fluid injection pressure^9^, rate^21^, viscosity^22^, volume^23^, and type^24^; and, the uncontrollable subsurface parameters, including stress state^12^ and pore pressure^25^, size and density of pre-existing faults/fractures^26^, fault/fracture orientation^26^ and frictional strength, steady-state coefficient of friction^27^ and rock’s permeability and compressibility^28^. However, wide inherent uncertainty affects the value of each uncontrollable parameter. In HF treatments, accounting for parametric uncertainty by using appropriate probability distributions^9,11^ leads to better decision-making for user-controlled parameters such as injection pressure. Because of large-scale injection-induced earthquakes in the Montney Formation, probabilistic fault slip assessment is essential to improve understanding of seismic hazards in the region. This is of importance because no studies on such a scale have been presented in the Montney Formation. Similar studies have been performed in Fox Creek, Alberta^29^, north-central Oklahoma^9^, the Fort Worth Basin^12^, and the Delaware Basin in Texas^11^.

This paper aims to assess the fault slip tendency resulting from fluid injection into the Montney Formation. Herein, we define a geomechanical zoning or stress area model based on pore pressure variation in the Montney Formation. We then assess all known faults as potential sites of injection-induced seismicity. In each stress area, we constrain uncertainties associated with each effective uncontrollable geomechanical parameter, such as stress tensors, pore pressure, multiple fault/fracture orientations, and frictional strengths. Then, we apply a probabilistic assessment to investigate the potential fault slip tendency due to HF in the formation, incorporating the uncertainty distributions associated with Mohr–Coulomb strength parameters. Besides the HF stimulations, the resulting probabilistic fault stability map in the region can be used as a baseline map for any fluid injection projects such as wastewater disposal, CO_2_ sequestration as well as geothermal energy extraction.

## State of stress in the Montney Formation

**Figure 2:**
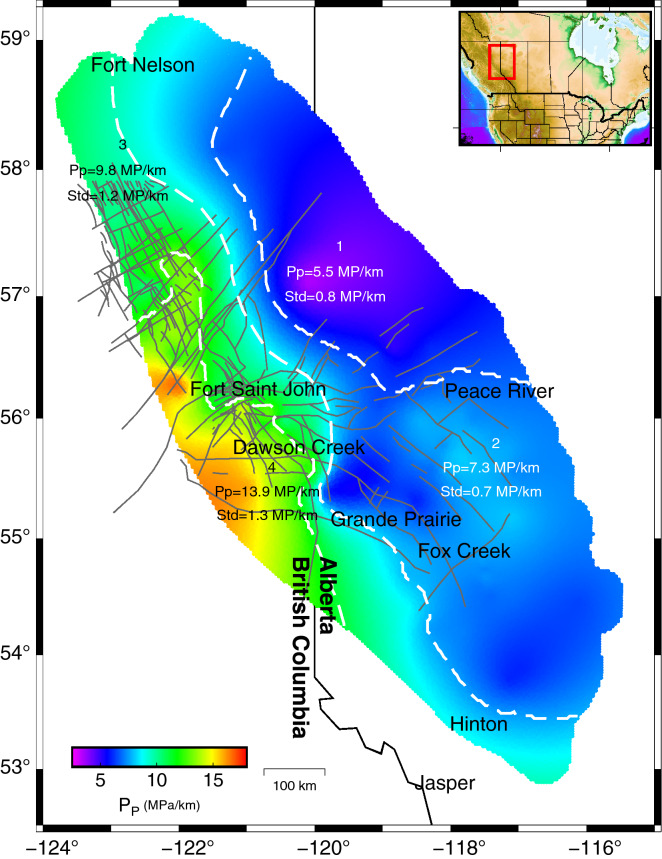
Spatial pore pressure gradient values in the Montney Formation. Extremely low Pp (5 MPa/km) are observed around Peace River and Grande Prairie where less seismicity has been recorded. Pore pressure gradients are highest (15 MPa/km) in the areas around Fort St. John and Dawson Creek. The western parts of the Formation have relatively higher pore pressure values and gradients than the eastern parts. Gray lines indicate faults crossing one another in the Montney play. The white dashes show the zoning of the Montney Formation based on pore pressure gradients at various locations. Each zone is represented by a mean pore pressure value (Pp) and a standard deviation (Std). The figure was generated using GMT V.6^37^.

Pore pressure is an integral part of the state of stress in a region. Different studies have shown that pore pressure distribution in the Montney Formation is hydrologically subdivided and, consequently, the formation is compartmentalized^30–32^. Figure 2 sets out the lateral pore pressure variation of the Montney Formation mapped from direct pore pressure measurements taken from datasets provided by geoLOGIC™ Systems and Wozniakowska and Eaton^33^. The study by Chatellier and Euzen^30,31^ shows that the Montney Formation pore pressure compartments are due to hidden faults that do not appear in the 3D seismic dataset but rather have been determined by analyzing drilling cuttings and gas compositions (gas chromatography) and Diagnostic Fracture Injection Test (DFIT) results. Spatial variations of the pore pressure gradient (Fig. 2) indicate that the deeper, western side of the formation (in British Columbia) has a higher value than the shallower, eastern side (in Alberta).

Based on pore pressure variation, we subdivided the Montney Formation into four different areas and used the K-means MATLAB™ function to group pore pressure gradient datasets. The main reason for subdividing the formation is three principal stress magnitudes are intrinsically linked to the pore pressure. Therefore, when the pore pressure is high, there is little difference between the three principal stresses. Due to the fact that pore pressure is an important parameter in fault stability assessment, and since our analysis is based on injection pressure, dividing the formation into distinct areas allows a more accurate fault assessment. Note that the clustering is solely based on the value of the pore pressure gradient. The existence of faults or other factors such as stratigraphic variations and oil compositional differences in reservoir compartments has not been considered for the clustering. For the purpose of our assessment, we used the pore pressure that corresponded most closely with each fault patch (Fig. 2).

**Figure 3:**
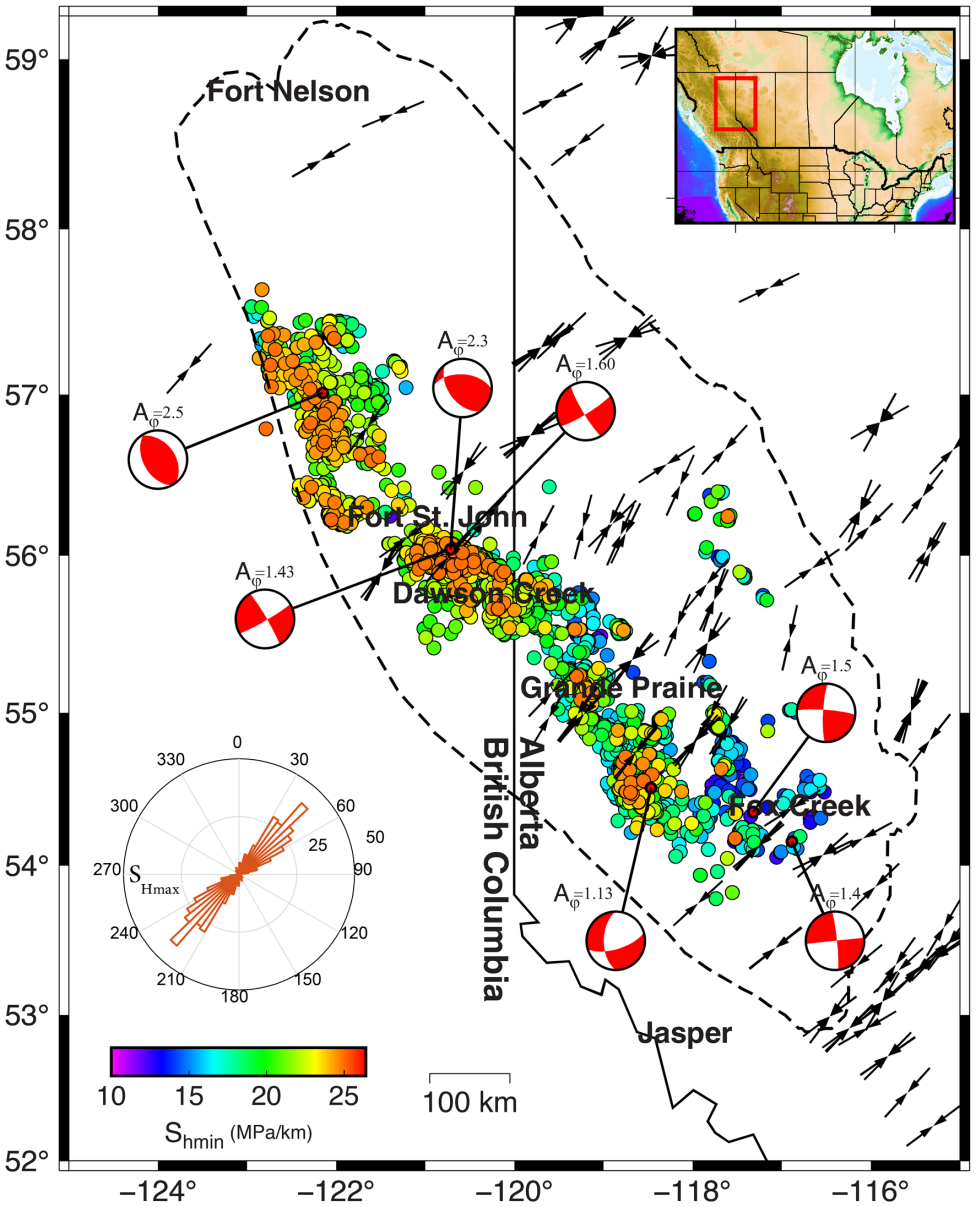
Map of S_hmin_ gradients in the Montney Formation. The data have been extracted from geoLOGIC™ systems. The black arrows, extracted from World Stress Map datasets^44^, represent the maximum horizontal principal stress (S_Hmax_) orientation (inset rose diagram). Beachballs present large-magnitude injection-induced focal mechanisms recorded in the studied area. This figure was produced using the GMT V.6^37^.

Comprehensive studies of principal stress orientations in British Columbia and Alberta have been conducted since the late 1970s^38–43^. Principal stress orientations in the region have been determined using different methods such as borehole failures (borehole breakouts and tensile-induced fractures) and earthquake focal mechanisms. Compilations of maximum horizontal compressive orientation (S_Hmax_) and relative stresses are available in the 2018 edition of the World Stress Map (WSM) databases^44^. Except around the Peace River Arch^41^, where S_Hmax_ is deflected because of the presence of complex fault systems, S_Hmax_ azimuth often strikes NE-SW in the region. Of 211 S_Hmax_ orientations provided by WSM in the study region, 19 have an A quality ranking, indicating that the dominant S_Hmax_ orientation is NE-SW. Enlighten Geoscience^45^ also determined fifty-eight (including 40 A quality data) S_Hmax_ orientations from borehole failures through various wellbores drilled in the Kiskatinaw area. The black arrows in Fig. 3 indicate the S_Hmax_ azimuth (including all quality rankings) within the region, derived from borehole breakouts and tensile-induced fractures provided by WSM and Enlighten Geoscience. Figure 3’s inset rose diagram, which includes all available datasets with different quality rankings, shows the consistency of S_Hmax_ orientations in the region. In this study, based on available borehole stress orientation indicators, we have assigned a mean of 45° and a standard division of 5° to S_Hmax_ azimuth in all stress areas. It should be pointed out that the stress orientation perturbation due to HF is assumed to be small relative to the regional stresses orientation.

The vertical stress (S_v_) is assumed to be equal to the average specific weight of the geomaterials multiplied by the depth. S_v_ can be obtained from the typical density logs that are abundant for most drilled wells. Because of density log availability, less uncertainty is associated with the vertical stress component in stress tensors. Several studies have investigated the vertical stress variation in the Western Sedimentary Basin^40,43,46,47^. Bell and Grasby^40^ showed that S_v_ varies between 22–25 MPa/km at a depth between 0.5 km and 1 km beneath the surface in the study area. The study of the Kiskatinaw area reported in Enlighten Geoscience^45^ indicates vertical stresses ranging from 24.6 to 25.5 MPa/km at the depth of the Montney Formation (~ 2.5 km). The same values were reported in Hayes et al.^48^ and Shen et al.^49^. In our study, we consider an S_v_ range of between 24 and 26 MPa/km.

Many wells drilled in the Montney Formation have undergone a Diagnostic Fracture Injection Test (DFIT) or mini-frac, which provides reliable determinations of minimum in situ stresses. In DFIT, closure pressure is considered to be a good estimate for minimum principal stress magnitudes (S_hmin_)^50^. Enlighten Geoscience^45^ after re-interpretating DFIT tests in the Kiskatinaw area provided by geoLOGIC systems, generated a map of the minimum principal stresses around Fort St John; S_hmin_ values were inferred to follow a normal distribution, ranging from 13.8 to 24 MPa/km, with a mean of 18.7 and standard deviations of 1.9 MPa. Using the closure pressure reported by Enlighten Geoscience^45^, along with operator-reported closure pressure gradients provided by geoLOGIC™ systems, Fig. 3 shows a map of the minimum stress magnitude gradients in the Montney Formation. In our study, we assume that the S_hmin_ gradients in the upper-middle Montney, lower-middle Montney, and lower Montney are the same. Figure S3 in Supplementary Information presents the map-inferred S_hmin_ gradients derived from the dataset provided in Fig. 3. Note that we also assume that the HF-induced stress perturbation and stress shadow effects are local and thus small relative to the regional stresses.

The spatial variations of the S_hmin_ gradients (Figs. 3 and Figure S3) indicate that minimum principal stress magnitudes are slightly higher on the British Columbia side than in Alberta, similar to the case for spatial pore pressure gradient values. A study of 134 DFITs in the Montney Formation indicated a direct relationship between pore pressure variation and S_hmin_ gradients^51^. Based on pore pressure zoning (Fig. 2) and the available minimum principal stress datasets (Fig. 3), we have derived statistical measures of the S_hmin_ magnitude variables in each stress area. In Supplementary information, Table S1 provides information about the mean and standard deviations of the Shmin gradients.

The maximum principal stress magnitude is the most difficult parameter to measure in a strike-slip (or thrust) stress state tensor. However, its range can be constrained by utilizing borehole failure data along with knowledge of minimum principal stresses and vertical stresses. Earthquake focal mechanisms also provide valuable information on the relative stress magnitudes and maximum principal stress magnitudes. In this study, we have used the injection-induced earthquake focal mechanisms recorded in WCSB to constrain the maximum principal stress magnitudes. The dataset includes 64 HF-induced earthquakes around Fort St John, and 39 wastewater-induced earthquakes near Musreau Lake, Alberta^35^.

One of the parameters that can be derived from the inversion of the focal mechanism is Angelier's shape parameter $\varphi = \frac{{S}_{2}-{S}_{3}}{{S}_{1}-{S}_{3}}$, in which S is the principal stress magnitude and S_1_ > S_2_ > S_3_. Simpson^52^ generalized the parameter $\varphi$ values to provide a quantitative measure with which to determine the relative stress magnitudes in each stress regime by expressing the equation as ${A}_{\varphi }=\left(n+0.5\right)+{\left(-1\right)}^{n}(\varphi -0.5)$ with n = 0, 1, 2, for normal, strike-slip and reverse faulting respectively. The Anderson fault parameter ${\mathrm{A}}_{\mathrm{\varphi }}$ ranges continuously from 0 to 1 for normal, 1 to 2 for strike-slip, and 2 to 3 for reverse faults^53–55^.

Applying Simpson’s approach to the combined 103 compiled focal mechanisms revealed that a strike-slip fault system is the dominant tectonic regime in the area, with an average Anderson fault parameter of ${A}_{\varphi }\approx 1.20$ around Musreau Lake and ${A}_{\varphi }\approx 1.7$ around Fort St John. Of the 103 focal mechanisms, 93 are strike-slip faulting events and the remainder are large-magnitude reverse faulting events that occurred in the Fort St. John Graben system^7^in the northern part of the study area. Roth et al.^7^ states that there is no obvious relationship between the faulting style events and hypocentral depth. Note that earthquake events recorded around Musreau Lake are not the result of injection operations in the Montney Formation but rather the result of injection at deeper depths in the Winterburn Formation^35^. However, injection-induced earthquakes that have occurred above and below the injection depth can provide valuable information on the region’s state of stress.

Figure 3 illustrates some of the focal mechanisms in the study area, with ${A}_{\varphi }$ representing the value above each beachball. In Supplementary Information, Table S3 lists the focal mechanism source data used in this study and the result of ${A}_{\varphi }$ on each focal mechanism. Applying the same approach to eleven earthquake focal mechanisms resulting from HF operations around Fox Creek, Yaghoubi et al.^56^ also concluded that ${A}_{\varphi }$ is 1.56 (strike-slip regime).

We constrain the magnitude of maximum horizontal principal stress, S_Hmax_, using ${A}_{\varphi }$. For stress area 4, for example, where the mean S_hmin_ and S_v_ gradients are 19.4 MPa/km and 25 MPa/km respectively, and the relative stress ratio is ${A}_{\varphi }\approx 1.67$, the ratio S_Hmax_/S_hmin_ is equal to 1.78. Consequently, the maximum horizontal stress gradients is around 34 MPa/km. The same value is assumed for stress area 3. For stress area 2, using Musreau Lake’s focal mechanism dataset, ${A}_{\varphi }$ is assigned a value of 1.2. Unfortunately, sufficient focal mechanisms for determining the relative stress ratio (${A}_{\varphi }$) are not available for stress area 1. Consequently, we assume a stress ratio $of {A}_{\varphi }\approx 1.2$ for this area.

## Fault

To evaluate fault slip assessment, we need information on the dip direction and dip angle. Most of the faults in the studied regions are hidden and completely buried under sedimentary rock units. Different studies have been performed to map faults in the region using high-resolution aeromagnetic (HRAM) data integrated with regional seismic, remote sensing, and drilled well information. The faults mapped in Fig. 1 are inferred and compiled from published studies, including those by Barclay et al.^57^, Berger^58^, Davies^59^, Davies^60^, Davies et al.^61^, Berger^62^, Berger et al.^63^, Furlong et al.^64^, and Hayes et al.^48^.

Since faults in the studied area are hidden, the three-dimensional geometry and dip angles are either unknown or are associated with uncertainties. However, the presence of seismicity in an area with a known state of stress provides useful information on seismogenic fault properties such as strike, dip direction, size, and the coefficient of friction. Considering the state of stress in the Montney Formation and slip compatibility analysis of 103 complied focal mechanisms, the hidden faults are expected to dip more than 60°. In this study, the dip angle of each fault is described as a probability distribution. Figure S4 in Supplementary Information presents a Mohr Diagram with a representative strike-slip focal event (Mw = 4.6, 2018–11–30) and resolved shear and normal stresses for each nodal plane. The nodal plane with high $\tau /{\sigma }_{n}$ is selected as the actual plane. These nodal planes are shown in bold in Table S3 in Supplementary Information. Aside from this, no laboratory studies or in-situ tests have been conducted to investigate the magnitude of coefficients of friction for regional faults. Based on experimental studies, Byerlee^63^ has shown that for different rock types, the coefficient of friction lies between 0.6 and 1. In our study, we assumed that the coefficient of friction ranges from 0.5 to 0.8. A similar value has been assigned to fault slip tendencies in Oklahoma^9^, the Delaware Basin of Texas, and New Mexico^11^. It should also be noted that some faults in this region have been mapped at slightly different locations in different studies. In this study, we mapped and considered both versions for slip assessment. Additionally, there are some areas where earthquakes are not associated with faults that have been mapped. Injection-induced earthquakes around the Fox Creek area, for example, are due to HF in parts of the Duvernay Formation that lie near critically stressed faults that had been unknown before the operation started^65^. Similarly, seismic activity around Musreau Lake has been linked to the reactivation of an unknown N-S fault due to wastewater injection at a depth of 3 to 4 km. These examples suggest that other areas with as-yet unrecognized critically stressed faults probably exist and will also be susceptible to HF-induced earthquakes.

## Assessment of fault-slip potential

Fault or fracture slip depends on the relative stress magnitude, the angle between the principal stress directions and the fault plane, and the coefficient of friction μ based on Coulomb faulting theory^10,66^. The slip tendency in a pre-existing cohesionless fault can be defined in terms of the Mohr–Coulomb shear failure criterion, where σ_n_ is the effective normal stress across the slip surface

1 $$\tau =\mu {\sigma }_{n}$$

Fault plane slippage is more likely to occur when the resolved shear stress, $\tau$, equals, or is very close to, the frictional resistance of the fault surface; the fault is then called “critically stressed”. The deterministic fault slip tendency is expressed as the ratio of effective normal stress to shear stress on a potential sliding surface ($\tau /{\sigma }_{n}\ge \mu$).

The deterministic approach considers just one single analysis as finite and therefore underestimates potential risks (see Figure S5 in Supplementary Information). The slip tendency in a probabilistic analysis, however, considers inherent uncertainties for each figure input variable, including stress magnitudes and orientations, fault dip directions, angles, and frictional strengths^9,67,68^. Each input variable effective in Mohr–Coulomb shear failure can be assigned as a random sample with specific statistical parameters. An appropriate probability distribution should be assigned for each of the uncertain input parameters in the model. The probability of failure can be defined as

2 $${P}_{f}=P\left[ \tau -\mu {\sigma }_{n}\le 0\right]$$

**Figure 4:**
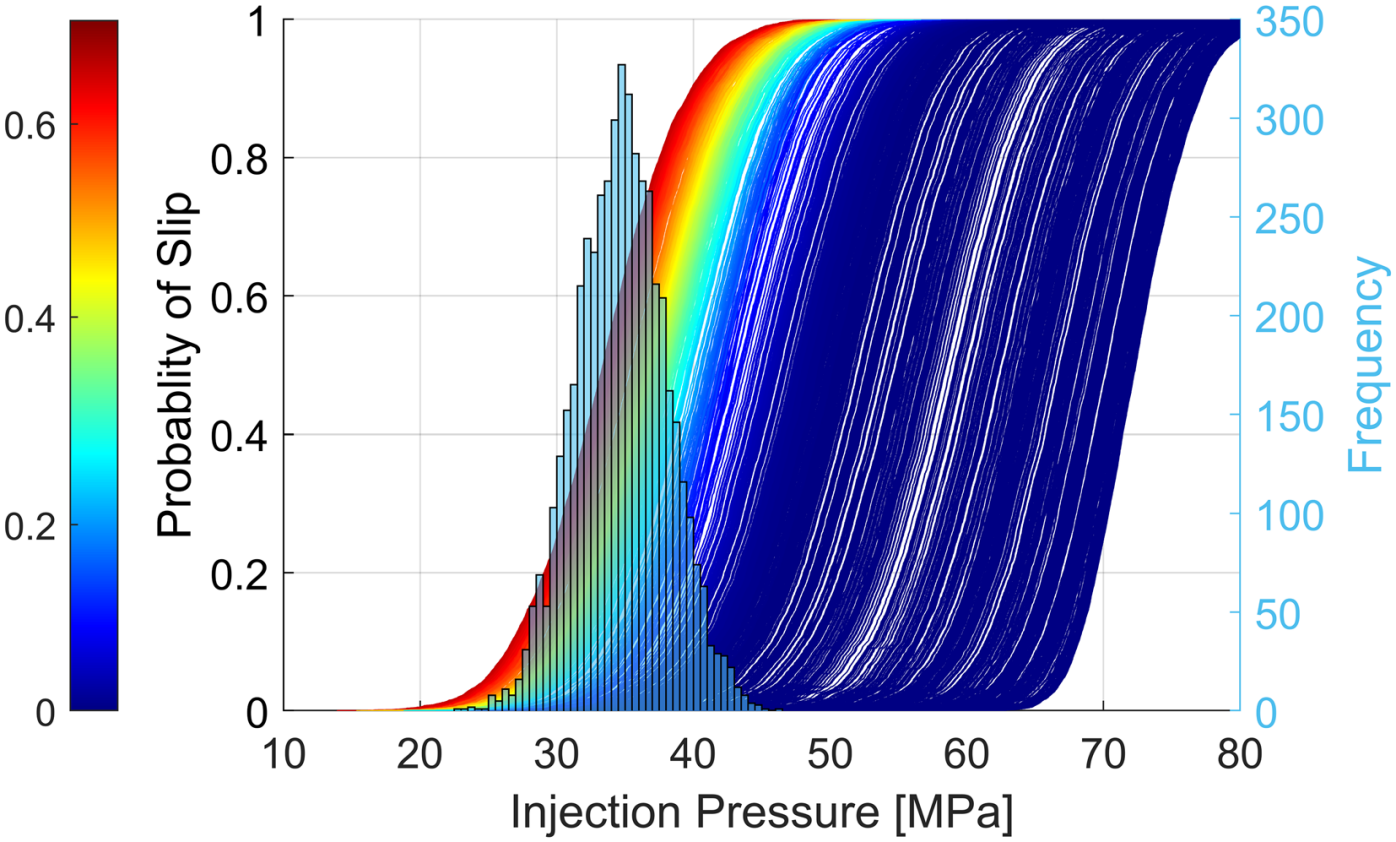
The cumulative probability function of the required injection pressure to cause slip on faults located in stress area 4. The histogram presents the pore pressure distribution in the stress area 4. Each curve represents the cumulative probability function of slip on each fault segment. The difference between current injection pressure and mean Pp distribution is 2 MPa. The depth of injection is assumed to be 2.5 km. This figure was produced using MATLAB™.

Probabilistic slip tendency analysis is, therefore, more comprehensive and more suitable for evaluating risk in multiple scenarios. In this study, for each fault patch, a Monte Carlo simulation with 5000 scenarios has been applied to evaluate the slip tendency of faults in the Montney Formation. In determining the size of the simulation sample, we considered the probability of fault slip (with two-digit precision) compared to the number of realizations (see Figure S6 in Supplementary Information). The analysis includes uncertainty associated with the uncontrollable subsurface parameters, such as the state of stress, pore pressure, pre-existing fault/fracture orientation, and frictional strength. Figures S7 to S10 show the statistical geomechanics variables used in the Monte Carlo simulation in each stress area. The result is a cumulative distribution of the probability of slip for each mapped fault. Figure 4 shows an example of the cumulative probability function of the injection pressure required to cause slip at a depth of 2.5 km in all fault segments located in stress area 4.

**Figure 5:**
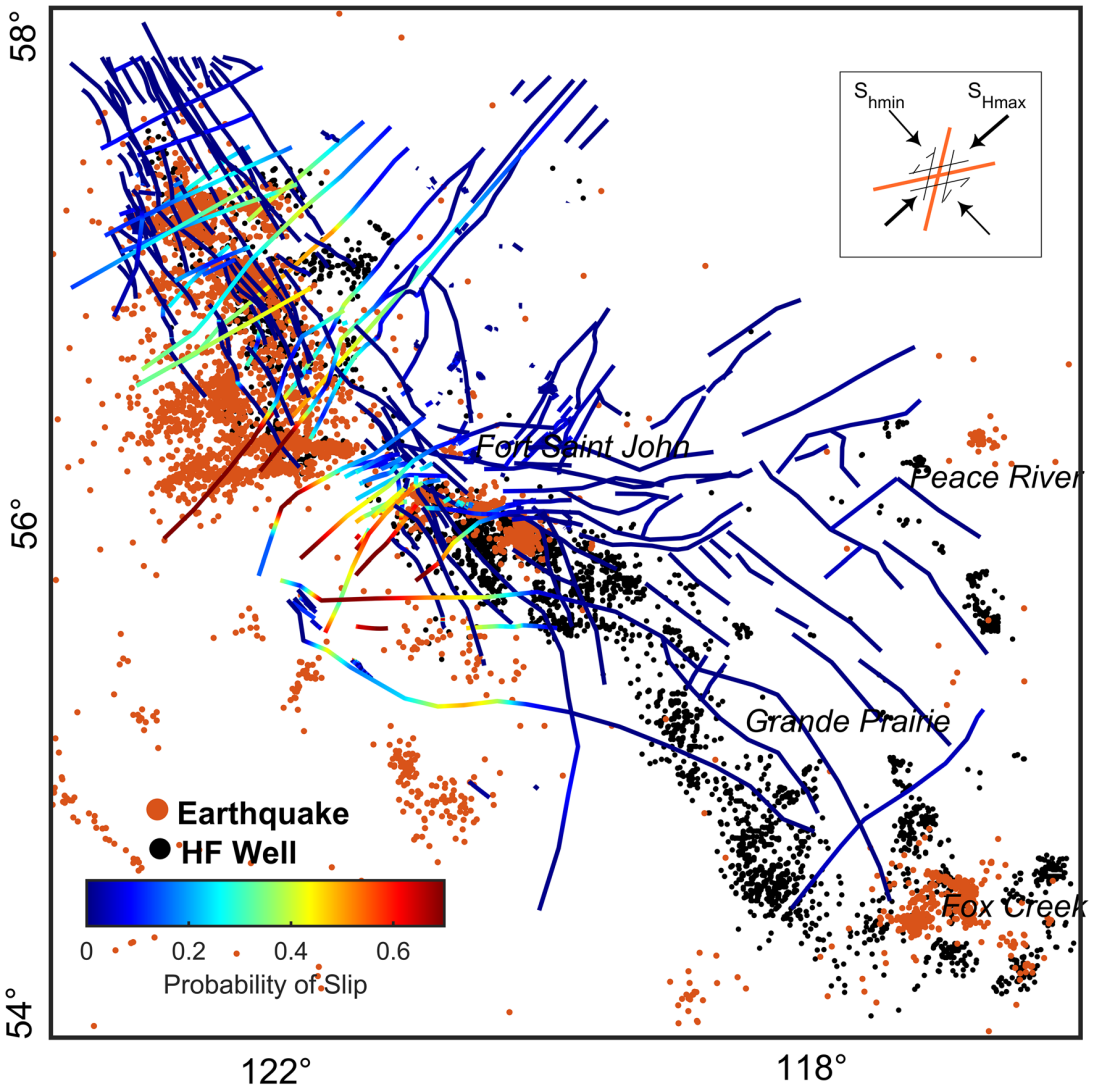
Fault map color-coded to highlight the probability of slip in the Montney Formation. Black points represent wells that were hydraulically stimulated. The red circles are seismicity reported by Visser, et al. 34 and Visser, et al. 8. This figure was produced using MATLAB™.

In Fig. 4, we use the uncertainty distributions in Figure S10 (in Supplementary Information) to apply Monte Carlo simulation to the faults mapped in study area 4. For this case, some segments of faults are likely to slip with an injection pressure of 37 MPa (at a depth of 2.5 km), and the probability of slip at the current injection pressure is 76%. The same analyses were performed for each fault patch mapped in Fig. 1 in different stress areas (see Figures S11, S12, S13). For each fault segment, we calculated the probability of slip in response to 2 MPa pore pressure perturbations (ΔP(P_inj_-P_p_) = 2 MPa) as presented in Fig. 4. Figure 5 shows faults mapped in the study area color-coded with the probability of slip. The red fault lines imply a higher likelihood of slip. Recorded earthquakes and wells drilled in the area are represented with black and red circles respectively.

## Discussion

The results of the study indicate that pore pressure gradient and fault orientation are important factors affecting seismic activity in the studied area. In overpressured areas, the principal stress magnitudes approach the vertical stress value regardless of the region’s fault regime environment. This fact is important in assessing fault/fracture stability because, in overpressured regions where the difference between the minimum and maximum principal stresses is smaller, a fault in the optimum orientation is likely to slip more easily. Of the 15,609 induced earthquakes presented by Visser et al.^34^ and Visser et al.^8^, only 13 are located in stress area 1, whereas more than 13,000 occurred in stress area 4, where the mean pore pressure gradient is 13.5 MPa/km. Around Grande Prairie, where the Montney Formation’s pore pressure gradient is around 7 MPa/km, no significant and large seismicity (M ≥ 4) has been reported even though more than 680 HF wells (represented by black circles in Fig. 5) have been stimulated with around 9 × 10^6^ m^3^ cumulative fluid injection (geoLOGIC™ Systems) into both the Duvernay and Montney Formations. As the seismic network in this area is sufficiently dense (Fig. 1), it can be concluded that there has been no seismic activity caused by fluid injection into the formations in the Grande Prairie area. This finding may be due to the relatively low pore pressure gradients in the Montney Formation or because pre-existing local faults are not in a critically stressed condition in the Grande Prairie area. However, Fox Creek (about 200 km southeast of Grande Prairie) is associated with large earthquakes, including one of M_W_ 4.1 on January 12, 2016, which has been associated with HF injected volume into the Duverney Formation^69^. Note that the mean pore pressure gradient in the Duverney Formation around Fox Creek is approximately 9 MPa/km greater than that in the Montney Formation in Grande Prairie. Thus, the importance of pore pressure gradient on induced seismicity in the Montney Formation is therefore evident.

Other factors in earthquake nucleation in the Montney Formation are the fault dip angle and dip direction. Slip compatibility analysis of 64 complied focal mechanisms (see Figure S3 in Supplementary Information) in the Kiskatinaw area shows that nodal planes optimal for slippage are expected to be in the Azimuth of 5° (or 185°) and 65° (or 245°). The observation is consistent with the frictional faulting theory and what we have illustrated in Fig. 5. The stereonet in Figure S5 in Supplementary Information shows fault slip tendency with an average S_Hmax_ orientation of 45° and ${A}_{\varphi }=1.7$ for stress area 4. The slip compatibility analysis of 39 focal mechanisms near Musreau Lake indicates a similar probability of slip between the two nodal planes (strike = 170 ± 20 dip = 60 ± 24 and strike = 85 ± 17 and dip = 65 ± 22); however, the nodal planes with a higher dip angle have a higher tendency to slip. In Fig. 5, the upper top inset shows the direction of a critically stressed fault.

This study is based on the mechanism by which earthquake nucleation occurs due to direct pore pressure diffusion along known faults in the Montney Formation. Similar studies have been performed in Fox Creek, Alberta^29^, north-central Oklahoma^9^, the Fort Worth Basin^12^, and the Delaware Basin in Texas^11^. Similar to finding for those areas^70,71^, injection-induced seismicity in the Montney Formations has been attributed to two other mechanisms as well: (a) poroelastic coupling stress evolution of the rock matrix between the injection zone and nearby fault^13^ and (b) an aseismic slip loading mechanism that causes delayed dynamic rupture events far from points of injection^18–20^.

Based on observations and evidence, all three mechanisms, individually or in combination, are plausible causes of earthquake nucleation in the Montney Formation at different locations of WCSB. However, the different mechanisms can be distinguished from one another by their spatiotemporal patterns of injection-induced seismicity. For example, the primary support for the aseismic loading mechanism is that most of the large events in WCSB are vertically offset from the injection zone and occur below (at crystalline basement depth) and above reservoir depth. In contrast to the aseismic loading mechanism, numerical stress modeling by Peña Castro, et al.^72^ has argued that highly permeable fault zones allow fluid from the injection zone to reach basement‐rooted faults in WCSB. The authors have indicated that rapid change in pore pressure along the fault is the dominant mechanism for the November 30, 2018 Mw 4.2 earthquake around Fort St John at a depth of 4.5 km, precipitated by HF in the Montney Formation (∼2.5 km depth). The existence of a permeable conduct/fault network is supported by a low flow rate for WCSB HF wells, as half of the injected fluid is lost during HF operations^73^.

Different effective parameters might be responsible for the various injection-induced seismicity mechanisms. Matrix permeability and compressibility are major factors in the poroelastic stress evaluation mechanism. Slow-slip-induced seismicity is likely to occur in shale-hosted faults with high clay and total organic content (TOC). However, the feature common to all three is the existence of critically stressed host faults in the region. In the context of Mohr–Coulomb failure criteria, as illustrated in the upper right inset in Fig. 5 and Figure S5, those faults striking ENE (Azimuth≈60° and 240°) and NEN (Azimuth≈10° and 190°) and dipping more than 60° are most likely to slip, as are the well-oriented fault planes in the studied area. The same analysis for reverse fault events shows the faults striking NWN and dipping from 15° to 60°have a high likelihood to slip (see Figure S14 in Supplementary Information). That finding is consistent with most of the reverse fault events in the study area. Even for those faults that are not critically stressed (where ${\sigma }_{n}\gg \tau$), fault reactivation and related induced seismicity can only be attributed to the aseismic loading mechanism. Hence, regardless of which mechanism is causing the significant anthropogenic seismicity in the region, this study provides information on known seismogenic faults in one of the largest unconventional shale gas resources in the world, the Montney Formation.

## Conclusions

We have used a probabilistic approach to determine the likelihood of fault slip as a function of injection pressure due to HF treatment in the Montney Formation. We first determined the state of stress and mapped faults as potential sites of injection-induced seismicity. The stress areas are defined by spatial pore pressure gradient variation. Strike-slip faulting regimes with ${\mathrm{A}}_{\mathrm{\varphi }}$=1.2 to 1.8 were determined using multivariable datasets from borehole petrophysical data to injection-induced focal mechanisms. Published known faults mapped for the Montney Formation were examined for slip tendency, considering the uncertainties associated with geomechanics parameters. Each geomechanical parameter was expressed as a probability distribution. Based on probabilistic analysis, it appears that most fault planes in the Kiskatinaw area and the northwestern Montney Formation would become unstable with only a moderate change in pore pressure. However, some areas have only a low probability of slip, having relatively low initial pore formation pressure. This finding is consistent with major injection-induced seismicity that has occurred in the area. In the Montney Formation, pore pressure spatial inhomogeneity plays a significant role in fault stability and injection-induced earthquakes. These results prompted us to discuss two important factors influencing fault stability in the Montney Formation: pore pressure gradient and fault direction. The areas with the highest pore pressure gradient and nearly vertical faults-oriented ENE (Azimuth≈60°) and NEN (Azimuth≈10°) are the most seismogenic regions in this unconventional play. The resulting probabilistic fault stability map can be used as a base map for fluid injection projects involving wastewater disposal, carbon sequestration and storage, and geothermal energy extraction.

## Supplementary Information

Supplementary Information.

## Data Availability

Focal mechanism data are included in the Supplementary Information. Pore pressure and minimum principal stress data are available from geoLOGIC™ systems. Other datasets that support the findings of this study are publicly available from the sources cited.
