# Supplementary material for: Injection-induced fault slip assessment in Montney Formation in Western Canada
Source: Sci Rep. 2022 Jul 7;12:11551. doi: 10.1038/s41598-022-15363-8 (PMC9262911; doi:10.1038/s41598-022-15363-8)
Supplement: Supplementary file 1 — Supplementary Information. [file 41598_2022_15363_MOESM1_ESM.docx]

Supporting information for

**Injection-Induced Fault Slip Assessment in Montney Formation in Western Canada**

Yaghoubi, A^1^, Dusseault, M.B^1, 2^, Leonenko, Y^1,3^

^1^*University of Waterloo, Department of Earth and Environmental Sciences, Waterloo, Ontario, Canada*

^2^*University of Waterloo, Waterloo Institute for Sustainable Energy (WISE), Waterloo, Ontario, Canada*

^3^*University of Waterloo, Department of Geography and Environmental Management, Waterloo, Ontario, Canada*

*Email: ali.yaghoubi@uwaterloo.ca*

Figure S1: Cumulative number of earthquakes with Mw >3 within areas 52°N to 60°N and 114°W to 126°W in Western Canada, indicating a rapid increase in the last decade.


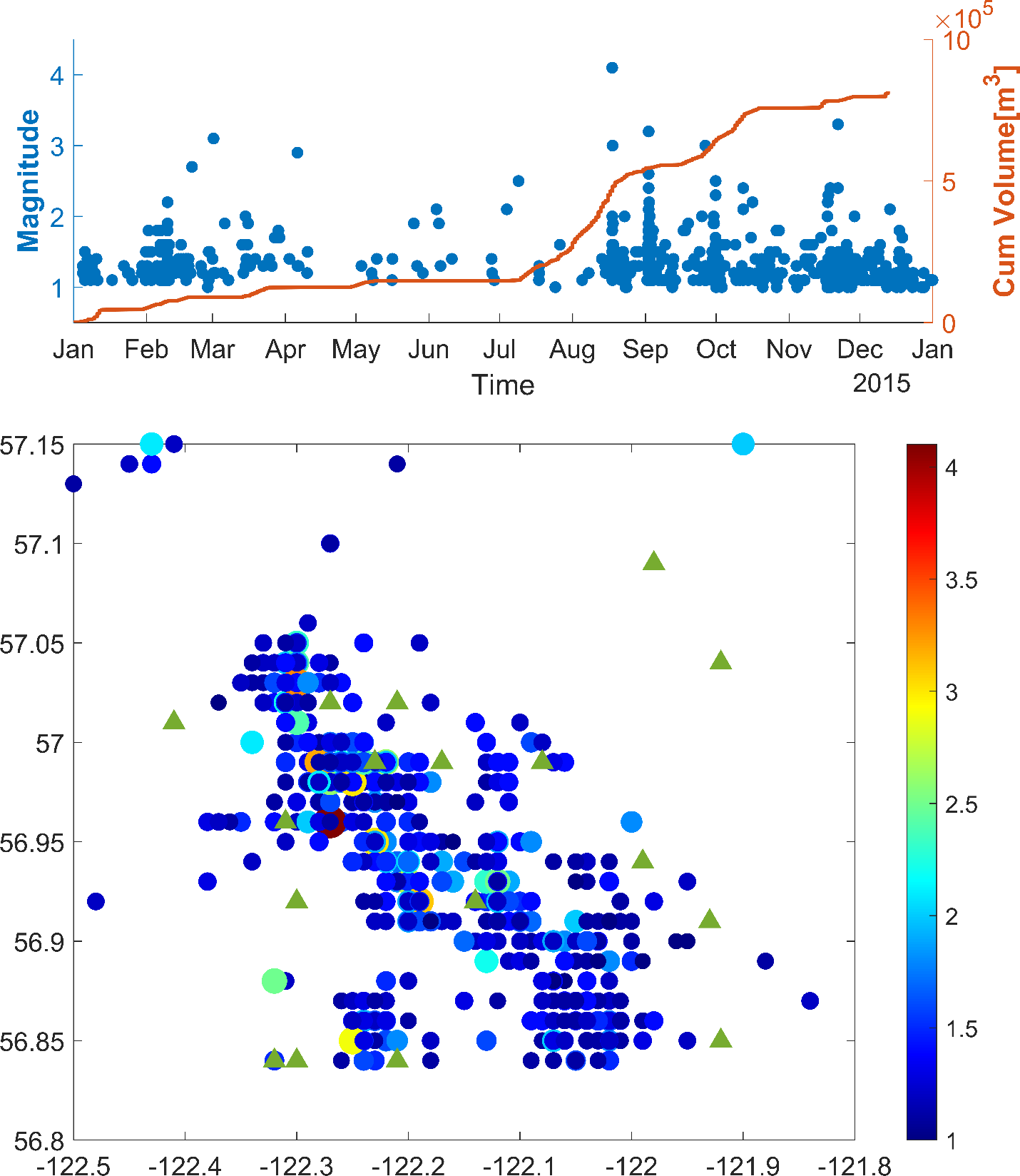


Figure S2:a) Approximately 8.2×10^5^ m^3^ of fluid has been injected in 16 wells in the Kiskatinaw area causing cumulative fluid injection and 617 earthquakes. b) Distribution of the local seismicity along with locations of the 16 wells.

Figure S3: Minimum horizontal stress gradient values in the Montney Formation, derived from the source dataset presented in Figure 3. This figure was produced using MATLAB^TM^.


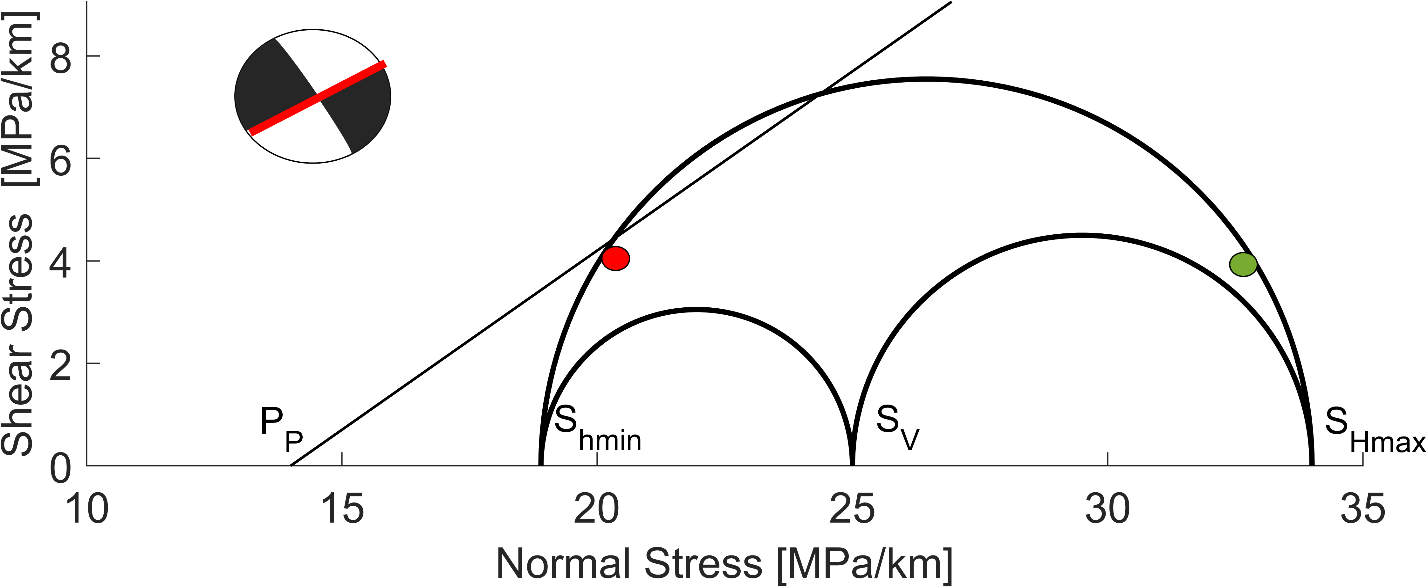


Figure S4: The Mohr-Coulomb shear failure criterion (the diagonal line) and 3D Mohr diagram 3D Mohr's circle representing a representative strike-slip earthquake focal plane (Mw=4.6, 2018-11-30). The red line on the focal beach ball indicates the actual fault plane.

Figure S5: Stereonet plot illustrates the slip-tendency (ratio of resolved shear to normal stress) for the strike-slip faulting regime ($A_{\varphi}\approx1.7$) in stress area 4. The red lines indicate the direction of the critically stressed faults in the Kiskatinaw area.

Figure S6: Probability of slip in a fault located in stress area 4 versus Monte Carlo simulations. 5000 represents an appropriate sample size (with two-digit precision) for Monte Carlo simulation for this study.


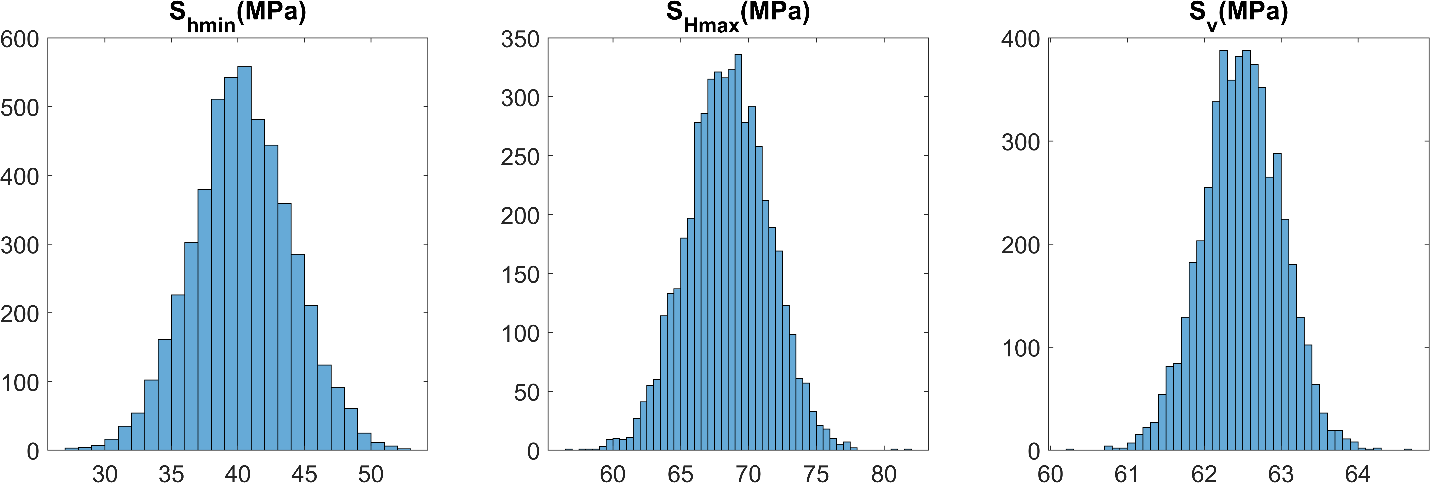


Figure S7: Variables used in Monte Carlo simulations for stress area 1.


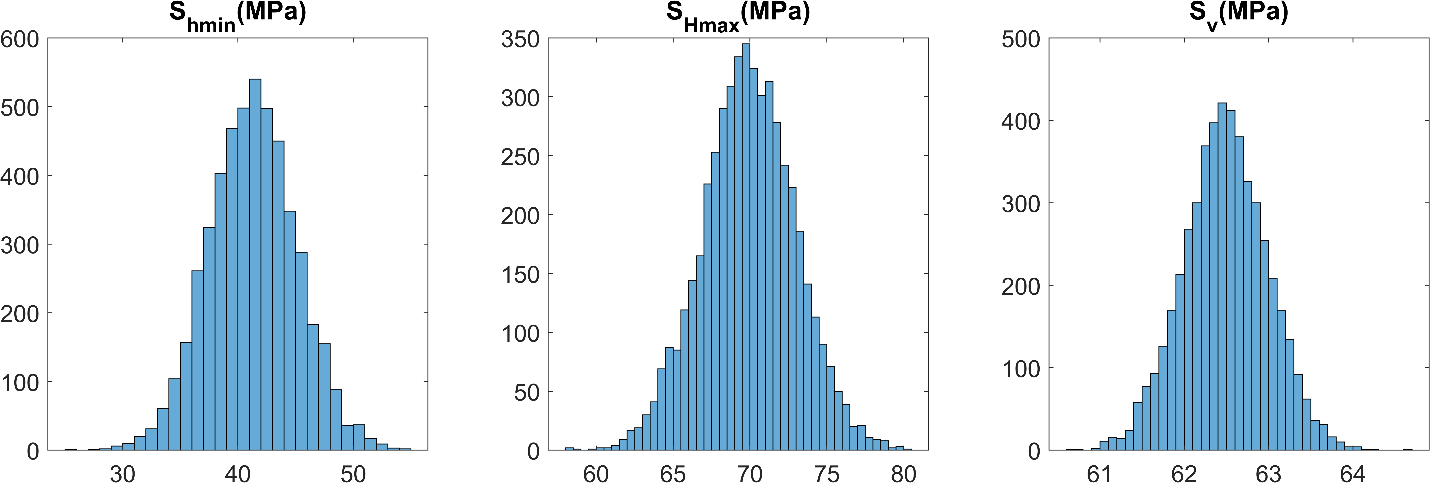


Figure S8: Variables used in Monte Carlo simulations for stress area 2.


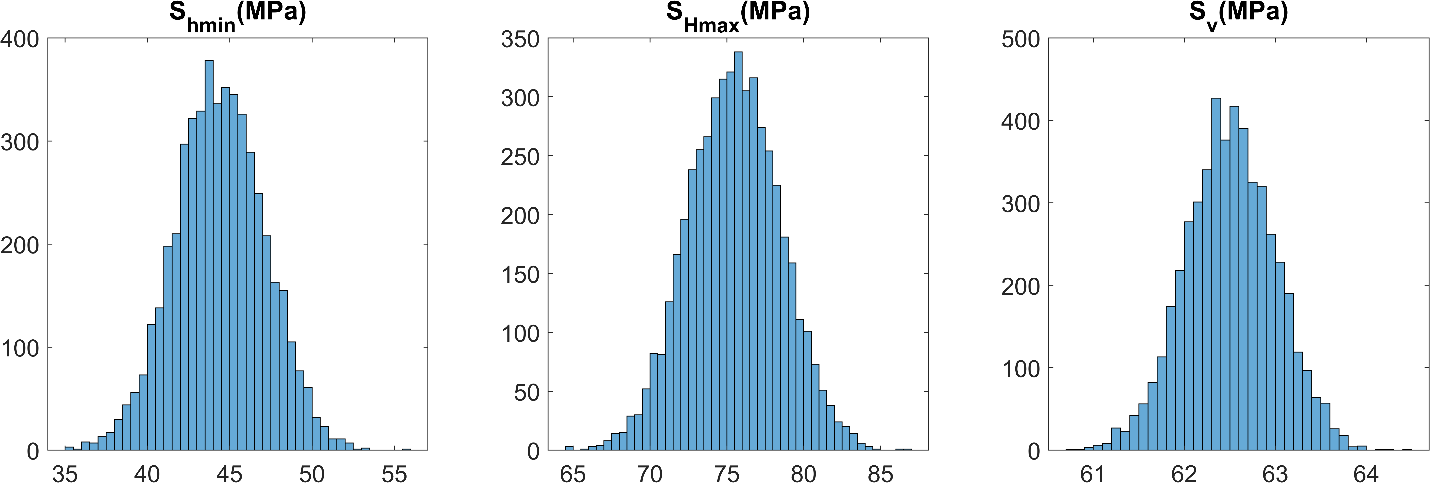


Figure S9: Variables used in Monte Carlo simulations for stress area 3.


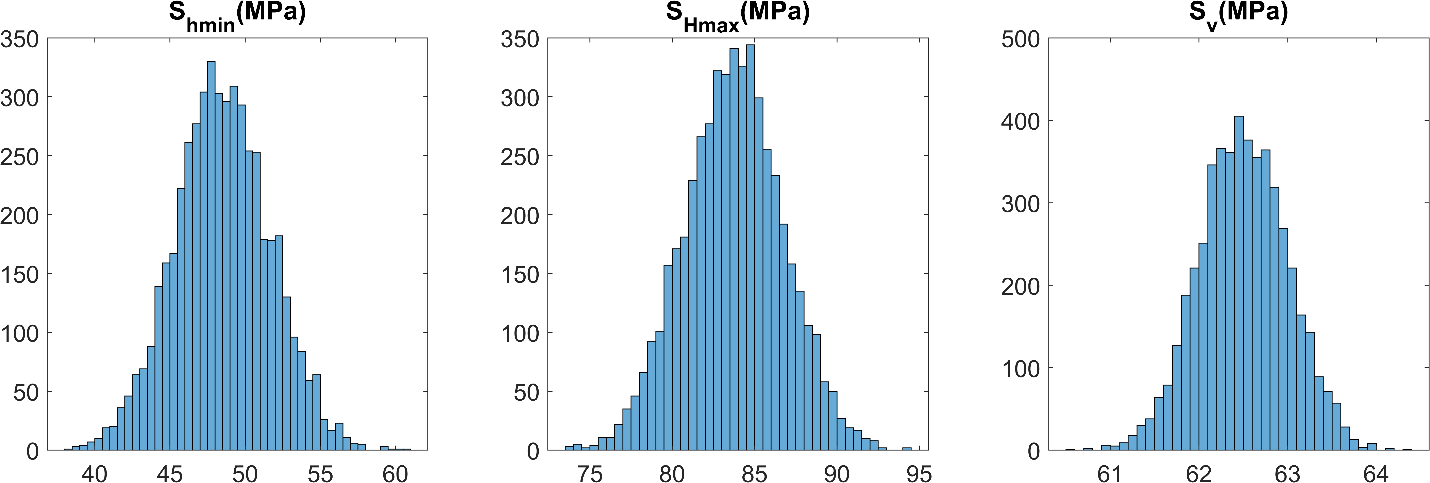


Figure S10: Variables used in Monte Carlo simulations for stress area 4.


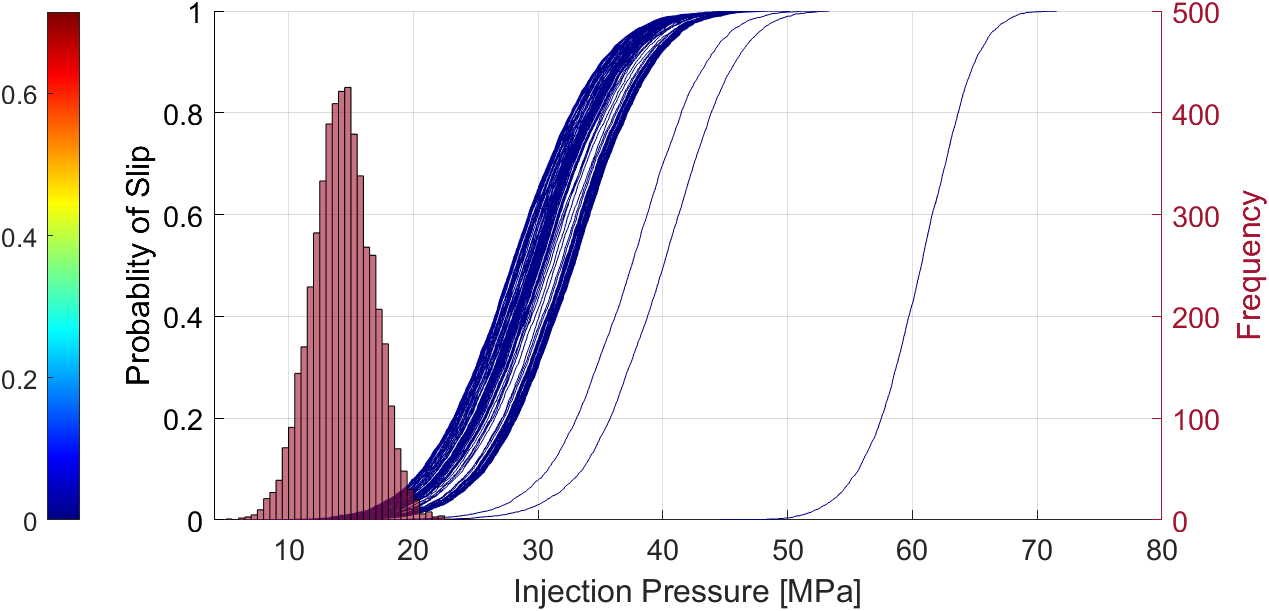


Figure S11: The cumulative probability function of the injection pressure required to cause a slip on faults located in stress area 1. A histogram illustrating the distribution of pore pressure in an area of stress 1. Each curve represents the cumulative probability function of slip for each fault segment at different injection pressure.


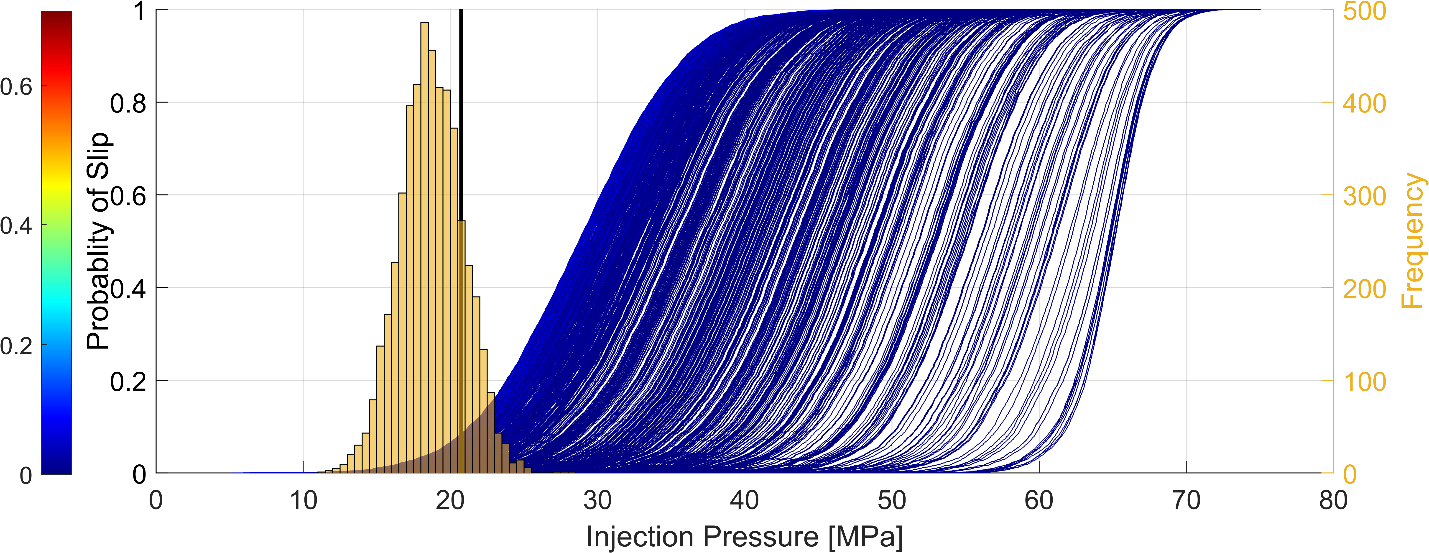


Figure S12: The cumulative probability function of the injection pressure required to cause a slip on faults located in stress area 2. The histogram illustrating the distribution of pore pressure in an area of stress 2.


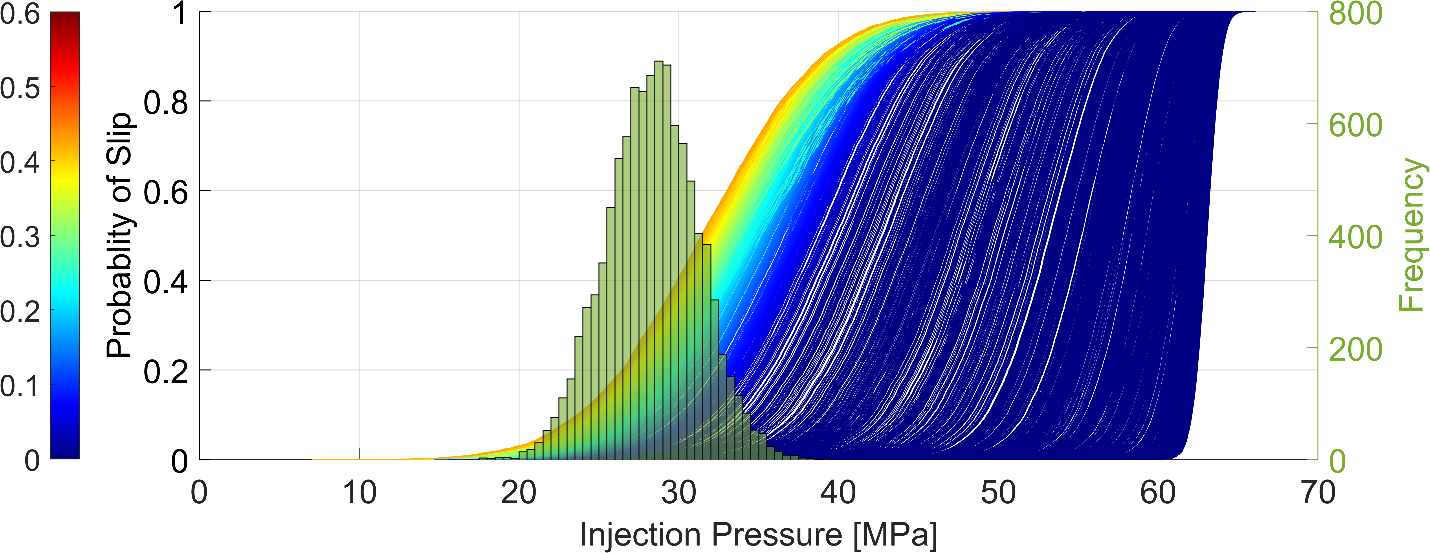


Figure S13: The cumulative probability function of the injection pressure required to cause a slip on faults located in stress area 3. The histogram illustrating the distribution of pore pressure in an area of stress 3.


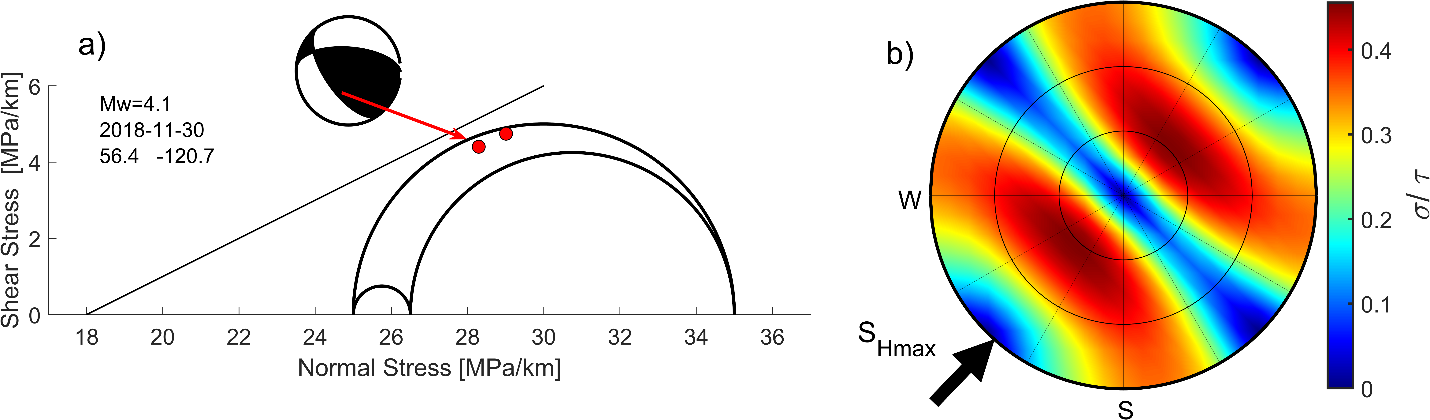


Figure S14. (a) The Mohr-Coulomb shear failure criterion (the diagonal line) and 3D Mohr's circle, depicting the reverse focal mechanism and resolved normal and shear stresses for each nodal plane. (b) Lower hemisphere stereonet plot for the case that state of stress is reverse faulting regime. Colors show the ratio of shear to effective normal stresses (required μ) needed for shear failure on a fault plane. In a reverse faulting regime, faults dipping from 15° to 60° and striking northwest to southeast are critically stressed

Table S1: S_hmin_ statistical measures are used in Monte Calor Simulation for each stress area. The number of earthquakes reported by Visser et al. (2017 and 2020) represents the number of occurrences in each stress area.

| **Stress Area** | **mean Shmin** | **Standard Deviation** | **Number of DFIT** | **Number of the earthquakes** |
| --- | --- | --- | --- | --- |
| 1 | 16.1 | 1.6 | 10 | 13 |
| 2 | 16.5 | 2.1 | 936 | 855 |
| 3 | 17.8 | 1.7 | 1742 | 1533 |
| 4 | 19.4 | 1.85 | 2299 | 13208 |

Table S2: S_v_ and S_Hmax_ statistical measures are used in Monte Carlo simulations for each stress area.

| **Stress area** | **Sv**  **(MPa/km)** | **Sv**  **Standard Deviation** | **S_Hmax_** | **S_Hmax_**  **Standard Deviation** | **Fault μ** | **Fault μ Standard Deviation** | **Fault**  **Dip** | **Fault**  **Dip** |
| --- | --- | --- | --- | --- | --- | --- | --- | --- |
| 1 | 25 | 1 | 27.4 | 2.7 | 0.65 | 0.05 | 75 | 4 |
| 2 | 25 | 1 | 28 | 2.5 | 0.65 | 0.05 | 75 | 4 |
| 3 | 25 | 1 | 30.2 | 3.1 | 0.65 | 0.05 | 75 | 4 |
| 4 | 25 | 1 | 34 | 3.1 | 0.65 | 0.05 | 75 | 4 |

Table S3. Earthquake source parameters used for determining stress and tectonic regime $(\mathbf{A}_{\boldsymbol{\varphi}})$in the Montney Formation. The possible/actual nodal planes (Strike, Dip, and Rake) are indicated in bold.

|  | **Lat** | **Lon** | **Strike** | **Dip** | **Rake** | **Strike** | **Dip** | **Rake** | $\mathbf{A}_{\boldsymbol{\varphi}}$ |
| --- | --- | --- | --- | --- | --- | --- | --- | --- | --- |
| 1 | 54.53 | -118.51 | **182.50** | **61.40** | **-147.10** | 75.29 | 61.52 | -33.00 | 1.02 |
| 2 | 54.53 | -118.51 | **179.80** | **69.20** | **-158.80** | 81.96 | 70.24 | -22.17 | 1.04 |
| 3 | 54.53 | -118.51 | **182.40** | **66.90** | **-157.80** | 83.30 | 69.66 | -24.73 | 1.12 |
| 4 | 54.53 | -118.51 | **179.50** | **65.20** | **-152.40** | 77.13 | 65.13 | -27.54 | 1.04 |
| 5 | 54.57 | -118.52 | **174.50** | **73.00** | **-172.30** | 82.24 | 82.64 | -17.15 | 1.26 |
| 6 | 54.56 | -118.53 | 185.00 | 69.70 | -170.20 | **91.57** | **80.81** | **-20.58** | 1.44 |
| 7 | 54.57 | -118.53 | **170.00** | **68.30** | **-161.60** | 72.99 | 72.95 | -22.75 | 1.05 |
| 8 | 54.56 | -118.54 | **181.20** | **62.80** | **-147.20** | 74.79 | 61.20 | -31.44 | 1.08 |
| 9 | 54.53 | -118.51 | 185.40 | 56.10 | -157.30 | **82.27** | **71.32** | **-36.07** | 1.33 |
| 10 | 54.56 | -118.54 | **177.60** | **69.00** | **-166.30** | 82.61 | 77.23 | -21.56 | 1.21 |
| 11 | 54.53 | -118.50 | **175.40** | **72.10** | **-162.80** | 79.97 | 73.66 | -18.68 | 1.00 |
| 12 | 54.53 | -118.51 | **172.50** | **48.50** | **-158.10** | 67.58 | 73.78 | -43.64 | 1.15 |
| 13 | 54.53 | -118.44 | **174.60** | **69.60** | **-146.00** | 71.37 | 58.39 | -24.16 | 1.35 |
| 14 | 54.53 | -118.42 | **191.80** | **56.00** | **-127.40** | 65.62 | 48.81 | -48.00 | 0.84 |
| 15 | 54.51 | -118.47 | 190.40 | 49.20 | -159.00 | **86.32** | **74.26** | **-42.76** | 1.50 |
| 16 | 54.51 | -118.46 | **176.80** | **50.10** | **-157.30** | 71.78 | 72.78 | -42.19 | 1.21 |
| 17 | 54.51 | -118.46 | **177.80** | **47.10** | **-157.40** | 71.98 | 73.65 | -45.19 | 1.24 |
| 18 | 54.51 | -118.47 | **180.20** | **55.50** | **-151.30** | 72.97 | 66.69 | -38.08 | 1.11 |
| 19 | 54.51 | -118.46 | **189.00** | **50.70** | **-158.50** | 84.99 | 73.52 | -41.34 | 1.46 |
| 20 | 54.50 | -118.48 | **174.00** | **74.90** | **-165.10** | 80.03 | 75.63 | -15.60 | 1.03 |
| 21 | 54.51 | -118.48 | **176.30** | **59.80** | **-154.10** | 72.57 | 67.82 | -32.90 | 1.04 |
| 22 | 54.51 | -118.49 | **177.20** | **67.90** | **-165.60** | 81.68 | 76.68 | -22.74 | 1.20 |
| 23 | 54.51 | -118.48 | 185.30 | 53.60 | -167.20 | **87.62** | **79.73** | **-37.09** | 1.50 |
| 24 | 54.51 | -118.46 | **348.50** | **80.40** | **170.60** | 80.08 | 80.73 | 9.73 | 1.87 |
| 25 | 54.50 | -118.46 | **172.00** | **65.20** | **-158.90** | 72.81 | 70.93 | -26.35 | 1.02 |
| 26 | 54.50 | -118.48 | **169.40** | **68.50** | **-164.00** | 73.40 | 75.14 | -22.28 | 1.02 |
| 27 | 54.56 | -118.53 | **170.00** | **88.10** | **-177.80** | 79.93 | 87.80 | -1.90 | 1.16 |
| 28 | 54.51 | -118.48 | **177.60** | **61.70** | **-163.40** | 79.56 | 75.43 | -29.33 | 1.23 |
| 29 | 54.51 | -118.48 | **165.90** | **72.30** | **-156.30** | 68.30 | 67.49 | -19.22 | 1.40 |
| 30 | 54.51 | -118.48 | **165.40** | **73.40** | **-157.60** | 68.68 | 68.58 | -17.87 | 1.40 |
| 31 | 54.51 | -118.48 | **170.30** | **68.40** | **-162.40** | 73.64 | 73.67 | -22.56 | 1.01 |
| 32 | 54.51 | -118.48 | **177.30** | **59.50** | **-159.90** | 76.78 | 72.78 | -32.10 | 1.18 |
| 33 | 54.52 | -118.46 | **173.60** | **64.10** | **-169.30** | 78.88 | 80.39 | -26.30 | 1.26 |
| 34 | 54.52 | -118.46 | 2.30 | 63.30 | 160.20 | **101.49** | **72.39** | **28.13** | 1.97 |
| 35 | 54.51 | -118.48 | **178.90** | **70.10** | **-161.00** | 82.22 | 72.17 | -20.95 | 1.07 |
| 36 | 54.53 | -118.49 | **160.70** | **84.20** | **-154.00** | 67.88 | 64.14 | -6.45 | 1.67 |
| 37 | 54.50 | -118.48 | **176.40** | **73.60** | **-173.90** | 84.67 | 84.15 | -16.49 | 1.34 |
| 38 | 54.51 | -118.48 | **177.70** | **63.10** | **-161.10** | 78.89 | 73.21 | -28.20 | 1.17 |
| 39 | 54.51 | -118.48 | **186.60** | **60.40** | **-152.30** | 82.06 | 66.16 | -32.68 | 1.21 |
| 40 | 55.99 | -120.62 | **68.73** | **85.31** | **1.72** | 338.59 | 88.29 | 175.31 | 1.79 |
| 41 | 55.99 | -120.71 | **72.92** | **56.84** | **158.70** | 174.95 | 72.30 | 35.04 | 1.18 |
| 42 | 56.00 | -120.66 | **255.32** | **79.34** | **29.16** | 159.43 | 61.39 | 167.83 | 1.62 |
| 43 | 55.98 | -120.29 | **251.26** | **86.39** | **1.78** | 161.14 | 88.23 | 176.39 | 1.81 |
| 44 | 55.99 | -120.70 | **240.96** | **72.28** | **-6.66** | 332.99 | 83.66 | -162.17 | 1.57 |
| 45 | 55.97 | -120.69 | **241.68** | **74.73** | **-5.07** | 333.02 | 85.11 | -164.67 | 1.57 |
| 46 | 55.90 | -120.37 | **67.02** | **86.47** | **-1.08** | 157.09 | 88.93 | -176.47 | 1.49 |
| 47 | 55.97 | -120.48 | **72.61** | **58.30** | **27.16** | 327.52 | 67.15 | 145.23 | 2.03 |
| 48 | 55.95 | -120.56 | **58.08** | **76.58** | **0.67** | 327.93 | 89.35 | 166.58 | 1.80 |
| 49 | 55.93 | -120.50 | **73.39** | **49.76** | **33.19** | 320.49 | 65.30 | 134.68 | 2.03 |
| 50 | 55.95 | -120.63 | **258.53** | **81.68** | **4.06** | 167.94 | 85.98 | 171.66 | 1.68 |
| 51 | 55.93 | -120.26 | **245.73** | **84.78** | **167.39** | 336.90 | 77.44 | 5.34 | 1.15 |
| 52 | 55.95 | -120.64 | **63.10** | **88.47** | **-1.17** | 153.13 | 88.83 | -178.47 | 1.35 |
| 53 | 55.91 | -120.26 | **249.89** | **87.20** | **18.36** | 158.96 | 71.66 | 177.05 | 1.48 |
| 54 | 55.94 | -120.31 | **245.30** | **75.47** | **2.47** | 154.68 | 87.60 | 165.46 | 1.75 |
| 55 | 56.01 | -120.71 | **243.96** | **86.84** | **-12.65** | 334.67 | 77.37 | -176.76 | 1.20 |
| 56 | 55.90 | -120.18 | **274.72** | **64.08** | **57.55** | 150.21 | 40.63 | 137.83 | 2.36 |
| 57 | 56.04 | -120.45 | **303.10** | **74.21** | **163.49** | 37.71 | 74.13 | 16.43 | 1.06 |
| 58 | 56.04 | -120.45 | **78.08** | **47.60** | **44.75** | 314.32 | 58.68 | 127.87 | 2.15 |
| 59 | 55.91 | -120.37 | **245.31** | **89.90** | **5.07** | 155.30 | 84.93 | 179.90 | 1.34 |
| 60 | 55.89 | -120.54 | **59.27** | **85.24** | **-12.59** | 150.33 | 77.45 | -175.12 | 1.08 |
| 61 | 55.91 | -120.56 | **63.25** | **88.91** | **3.93** | 333.17 | 86.07 | 178.90 | 1.43 |
| 62 | 56.05 | -120.95 | 101.05 | 69.31 | 1.44 | **10.55** | **88.66** | **159.30** | 1.13 |
| 63 | 56.04 | -120.58 | **243.98** | **86.50** | **12.76** | 153.18 | 77.26 | 176.41 | 1.43 |
| 64 | 55.93 | -120.56 | **62.07** | **88.77** | **0.38** | 332.06 | 89.62 | 178.77 | 1.86 |
| 65 | 56.04 | -120.68 | **253.92** | **71.87** | **10.55** | 160.60 | 79.98 | 161.58 | 1.82 |
| 66 | 55.93 | -120.31 | **69.46** | **82.95** | **-11.14** | 160.84 | 78.94 | -172.82 | 1.10 |
| 67 | 55.84 | -120.55 | **64.86** | **78.16** | **-7.66** | 156.44 | 82.50 | -168.06 | 1.37 |
| 68 | 55.91 | -120.44 | **64.45** | **75.64** | **2.04** | 333.95 | 88.02 | 165.63 | 1.75 |
| 69 | 56.00 | -120.38 | **72.38** | **70.64** | **-4.73** | 163.95 | 85.54 | -160.58 | 1.42 |
| 70 | 55.99 | -120.61 | **64.62** | **82.61** | **8.31** | 333.54 | 81.76 | 172.54 | 1.73 |
| 71 | 55.93 | -120.36 | **247.02** | **89.28** | **-3.66** | 337.06 | 86.34 | -179.27 | 1.27 |
| 72 | 56.00 | -120.70 | **68.03** | **83.60** | **12.76** | 336.58 | 77.32 | 173.44 | 1.61 |
| 73 | 55.99 | -120.70 | **242.83** | **87.76** | **-0.61** | 332.86 | 89.39 | -177.76 | 1.58 |
| 74 | 56.05 | -120.70 | **261.08** | **57.65** | **32.74** | 152.10 | 62.82 | 143.02 | 2.02 |
| 75 | 56.04 | -120.70 | **252.24** | **66.34** | **33.93** | 147.13 | 59.25 | 152.17 | 2.28 |
| 76 | 56.04 | -120.69 | **271.08** | **68.54** | **54.64** | 153.81 | 40.63 | 145.81 | 2.46 |
| 77 | 56.04 | -120.69 | 125.73 | 66.51 | 107.83 | **266.83** | **29.19** | **54.84** | 2.04 |
| 78 | 55.91 | -120.39 | **68.58** | **79.93** | **9.30** | 336.94 | 80.84 | 169.80 | 1.90 |
| 79 | 55.89 | -120.38 | **66.39** | **78.75** | **-2.45** | 156.87 | 87.60 | -168.74 | 1.54 |
| 80 | 56.04 | -120.72 | 119.80 | 51.98 | 110.13 | **269.05** | **42.30** | **66.24** | 2.38 |
| 81 | 56.01 | -120.51 | **63.39** | **81.24** | **8.47** | 332.09 | 81.63 | 171.15 | 1.76 |
| 82 | 56.00 | -120.60 | **246.05** | **76.04** | **-3.54** | 336.91 | 86.56 | -166.02 | 1.53 |
| 83 | 55.99 | -120.62 | **244.58** | **62.60** | **25.09** | 142.42 | 67.88 | 150.22 | 1.68 |
| 84 | 55.98 | -120.46 | **66.73** | **71.61** | **2.19** | 336.04 | 87.92 | 161.60 | 1.70 |
| 85 | 56.07 | -120.94 | **280.42** | **75.12** | **30.09** | 181.95 | 61.02 | 162.92 | 1.82 |
| 86 | 55.90 | -120.36 | **249.69** | **82.37** | **5.08** | 159.01 | 84.96 | 172.34 | 1.92 |
| 87 | 55.93 | -120.55 | **65.78** | **75.50** | **-2.71** | 156.46 | 87.38 | -165.48 | 1.57 |
| 88 | 55.97 | -120.68 | **72.75** | **86.84** | **-8.11** | 163.20 | 81.90 | -176.81 | 1.27 |
| 89 | 56.04 | -120.65 | 68.60 | 83.16 | 14.67 | **336.81** | **75.44** | **172.93** | 1.60 |
| 90 | 55.99 | -120.65 | **91.99** | **88.47** | **-6.99** | 182.18 | 83.02 | -178.46 | 1.69 |
| 91 | 55.91 | -120.30 | **250.63** | **88.51** | **0.57** | 160.61 | 89.43 | 178.51 | 1.76 |
| 92 | 56.01 | -120.63 | **315.63** | **71.33** | **88.18** | 141.29 | 18.76 | 95.37 | 2.75 |
| 93 | 55.89 | -120.65 | **68.46** | **88.33** | **-6.51** | 158.65 | 83.49 | -178.32 | 1.26 |
| 94 | 55.93 | -120.27 | **67.38** | **87.81** | **-9.13** | 157.73 | 80.87 | -177.79 | 1.26 |
| 95 | 55.89 | -120.39 | **67.43** | **84.82** | **10.12** | 336.51 | 79.92 | 174.74 | 1.61 |
| 96 | 55.94 | -120.54 | **62.25** | **85.98** | **13.08** | 331.32 | 76.95 | 175.87 | 1.41 |
| 97 | 55.99 | -120.65 | **270.39** | **79.66** | **14.91** | 177.65 | 75.33 | 169.31 | 1.88 |
| 98 | 55.90 | -120.22 | **251.44** | **86.71** | **19.88** | 160.25 | 70.15 | 176.50 | 1.51 |
| 99 | 55.95 | -120.38 | **64.29** | **89.00** | **10.05** | 334.12 | 79.95 | 178.98 | 1.36 |
| 100 | 55.98 | -120.27 | **248.56** | **82.93** | **13.65** | 156.85 | 76.45 | 172.72 | 1.62 |
| 101 | 55.99 | -120.59 | **250.98** | **88.67** | **-2.36** | 341.03 | 87.64 | -178.67 | 1.15 |
| 102 | 55.94 | -120.66 | **71.50** | **79.60** | **-11.65** | 163.63 | 78.54 | -169.39 | 1.01 |
| 103 | 56.00 | -120.47 | **70.92** | **83.06** | **12.14** | 339.43 | 77.95 | 172.91 | 1.70 |
